# What are the surgical and oncologic outcomes, as well as the complications, associated with pre-operative radiotherapy in patients with low-grade soft-tissue sarcoma?

**DOI:** 10.1007/s10147-026-03059-2

**Published:** 2026-05-27

**Authors:** Hisaki Aiba, Joan Wang, Tsuyoshi Mizuma, Gerard Powell, Peter Choong, John Slavin, Claudia Di Bella

**Affiliations:** 1 Department of Orthopedics, Sarcoma Unit, St. Vincent’s Hospital Melbourne, 55 Victoria Parade, Fitzroy, Melbourne, VIC 3065 Australia; 2 Department of Orthopaedic Surgery, Nagoya City University Graduate School of Medical Sciences, Nagoya, Japan; 3 Department of Pathology, St. Vincent’s Hospital Melbourne, Melbourne, Australia; 4 Department of Orthopaedic Surgery, Keio University, Tokyo, Japan; 5 Department of Surgery, University of Melbourne, Melbourne, Australia

**Keywords:** Surgery, Low-grade sarcoma, Soft-tissue sarcoma, Radiotherapy, Plastic surgery

## Abstract

**Background:**

Surgery with radiotherapy is a standard choice for high-grade soft-tissue sarcoma; however, the indication of radiotherapy for low-grade soft-tissue sarcoma remains controversial due to an inherent low risk of distant metastasis and recurrence after surgery, as well as potential complications after surgery.

**Methods:**

Between 2007 and 2020, a total of 132 patients with low-grade soft-tissue sarcoma treated with pre-operative radiotherapy followed by surgical resection were examined. Pre-operative radiotherapy was administered with 50.4 Gy in 1.8 Gy fractions, with definitive surgery performed 4–8 weeks after completing pre-operative radiotherapy to allow inflammation to subside. Optimal methods for wound closure were performed by plastic surgeons, with the selective use of flap reconstructions (pedicled or free), skin grafts or direct closure.

**Results:**

Diagnoses included well-differentiated liposarcoma/atypical lipomatous tumors (*n* = 66), myxoid liposarcoma (*n* = 31), leiomyosarcoma (*n* = 22), and others. After en-bloc (wide) resections, 78.8% (104/132) underwent plastic reconstruction, including free flaps (51.5%), pedicled flaps (25.8%), and skin grafts (1.5%). The resection margins were R0 in 93.2% (123/132), R1 or R2 in 6.8% (9/132). The 5-year local recurrence-free rate was 99.1%, and distant metastasis-free survival was 90.6%, with nine metastases observed, mainly in myxoid liposarcoma. The disease-specific survival at 5 years was 99.2%. Wound complication-related reoperations occurred in 21.2% (28/132), with similar rates between direct closure (21.4%, 6/28) and plastic reconstruction (21.2%, 22/104, *p* = 0.705).

**Conclusion:**

Although surgical and oncological outcomes were favourable, the efficacy and invasiveness of pre-operative radiotherapy should be carefully balanced based on the patients’ individual background, due to the potential post-operative complications.

## Introduction

Soft-tissue sarcomas encompass a heterogeneous group of rare tumors with a mesenchymal origin [1]. Tumor grades correlate with a tumor’s aggressive behavior, serving as predictors of local recurrence and distant metastasis [2]; low-grade soft-tissue sarcomas exhibit lower risks of local recurrence and distant metastasis [1]. To improve local control, wide surgical resection combined with radiotherapy has become a standard choice for intermediate- to high-grade soft-tissue sarcomas [3]. Multimodal treatment strategies aim to achieve local control while preserving function and improving oncologic outcomes [1].

Radiotherapy for soft-tissue sarcoma has the potential to compromise wound healing, and may even worsen the limb function after surgery [1]. A prospective study of pre-operative radiotherapy for soft-tissue sarcomas reported a major complication rate of 35% within 120 days after surgery among 94 patients [4]. To minimize wound complications after surgery with pre-operative radiotherapy, the use of appropriate plastic reconstructions for the coverage of critical structures and dead space after tumor resection are considered to be important [5]. A retrospective study of 73 patients with soft-tissue sarcomas in extremities treated with pre-operative radiotherapy revealed that reoperations occurred in 26% of the cohort, but this rate decreased to 11% with plastic surgeon-assisted wound closure [6]. Thus, meticulous pre-operative planning and multidisciplinary collaboration in the resection and reconstruction are paramount to decrease the risk of peri-operative complications [7].

The rationale for this study is to elucidate whether pre-operative radiotherapy can further improve surgical and oncologic outcomes in patients with low-grade soft-tissue sarcomas, which are already associated with a low risk of local recurrence and distant metastasis. There remains a gap in evidence regarding the actual indication of pre-operative radiotherapy for low-grade soft-tissue sarcomas, as the potential oncological benefits need to be balanced against the risk of peri-operative complications. At our institution, we have traditionally performed pre-operative radiotherapy followed by wide surgical resection for all grades of soft-tissue sarcomas [8], based on the assumptions that pre-operative radiotherapy can improve oncological outcomes; relying on close multidisciplinary collaboration among orthopedic oncology and plastic reconstructive surgical teams to mitigate wound complications after pre-operative radiotherapy. Therefore, in this study, we retrospectively analyzed the outcomes of patients with low-grade soft-tissue sarcomas treated with pre-operative radiotherapy, with a focus on surgical and oncologic outcomes as well as wound complications.

## Patients and methods

### Study design and setting

This study is a retrospective, single-arm study conducted at a single specialized tertiary medical hospital. This study was approved by an ethical review board in our institution (approval no.: ERM109654). The requirement for informed consent was waived by the ethical review board because of the retrospective nature of the study. The procedures followed the ethical standards based on the Helsinki Declaration of 1975, as revised in 1983.

### Participants/study subjects

We identified 967 patients with all grades of soft-tissue sarcomas who were surgically treated at a single sarcoma center between January 2007 and June 2020. The exclusion criteria were: intermediate-to-high-grade soft-tissue sarcomas (*n* = 543); primary sites in the retroperitoneum, chest cavity, abdominal cavity, or head–neck area (*n* = 111); having undergone amputation (*n* = 66); tumor of cutaneous origin (*n* = 43); solitary fibrous tumors (*n* = 32); metastases before definitive surgery (*n* = 5); and a short follow-up (< 2 year; *n* = 25). Finally, 132 patients with low-grade soft-tissue sarcomas were analyzed (Table 1).

**Table 1:** Patients’ characteristics

Character (*n* = 132)		Number	Percentage
Tumor grade	Grade 1	132	100%
Gender (Female/Male)	Female	60	45.5%
	Male	72	54.5%
Histology	WDLS/ALT	66	50.0%
	Myxoid liposarcoma	31	23.5%
	Leiomyosarcoma	22	16.7%
	Myxofibrosarcoma	4	3.0%
	Low-grade fibromyxoid sarcoma	3	2.3%
	Others^*^	6	4.6%
Position	Upper extremity	25	18.9%
	Lower extremity	88	66.7%
	Trunk	19	14.4%
Size	Under 5 cm	67	50.8%
	Over 5 cm	65	49.2%
Depth	Superficial	17	12.9%
	Deep	115	87.1%
Previous surgery	No	92	69.7%
	Intralesional (unplanned) excision	37	28.0%
	Marginal excision	3	2.3%
Age (mean, standard deviation, range; year)	57.1	15.3	15.0-87.5

WDLS/ALT, well-differentiated liposarcoma/ atypical lipomatous tumor

*Others indicate unspecified spindle cell sarcoma (2 patients), unspecified myxoid sarcoma (1 patient), fibrosarcomca (1 patient), extraskeletal chondrosarcoma (1 patient) and malignant peripheral nerve sheath tumor (1 patient)

### Description of experiment, treatment or surgery

Diagnosis–All available histological slides of the resected specimens were reviewed and graded according to the Fédération Nationale des Centres de Lutte Contre le Cancer grading system. Regarding myxoid liposarcoma, grading was based on the area of the round cells, comprising a high nuclear-to-cytoplasmic ratio: if the area represented < 5% of the tumor, the myxoid liposarcoma was considered low-grade [9]. The diagnosis of well-differentiated liposarcoma/atypical lipomatous tumor (WDLS/ALT) was based on amplification of MDM-2 in fluorescent in situ hybridization analysis [10], and/or the presence of lipoblast or mature adipocytes with substantial variations in cell size and nuclear atypia in fatty or stromal spindle cells [11]. Based on the Union for International Cancer Control classification, the surgical margins of the definitive surgery were classified as microscopically negative (≥ 1 mm of normal tissue between the tumor and inked resection margin, R0), macroscopically negative but microscopically positive (minimal margin within 1 mm, R1), or grossly positive (R2) [12]. The extent of histological necrosis induced by pre-operative radiotherapy was calculated in percentage as the presence of neoplastic cells divided by the presence of viable atypical cells [13].

Treatment strategy–After confirming the diagnosis, treatment strategies for the patients are determined at our sarcoma multidisciplinary team meeting. As a standard approach, patients with soft-tissue sarcomas — irrespective of tumor grade — receive pre-operative external-beam radiotherapy (50.4 Gy in 1.8 Gy fractions). The radiotherapy fields are determined based on the gross tumor volume (the tumor and areas at risk, e.g. peri-tumor edema, hematoma, and biopsy tract) and clinical target volume (gross tumor volume plus a 3 cm longitudinal margin and a radial margin to encompass the fascial compartment) [7]. Definitive surgery is performed 4–8 weeks after completing pre-operative radiotherapy to allow inflammation to subside. Wide (en-bloc) resection is attempted for all patients. If sufficient surgical margins are not secured, the requirement of additional resection is discussed in the multidisciplinary meeting. In the pre-operative meeting between orthopedic oncology and plastic reconstructive team, optimal methods for wound closure are carefully discussed and planned. The selective use of flap reconstruction (pedicled or free) or skin grafts is determined based on appropriate coverage of critical neurovascular structures, range of the skin defect, tightness of the wound, and anatomical feasibility of the reconstruction.

### Aftercare

Postoperatively, the wounds are kept clean and dry, with regular dressing changes. Patients who undergo direct closure receive an occlusive dry dressing for 2–3 weeks. For patients who undergo flap reconstruction, soft, non-adhesive coverage is applied, and the wounds are closely monitored during the first seven days after surgery to assess flap viability. Anticoagulants are routinely used in patients, regardless of the type of wound closure. Joint movement is generally permitted with appropriate limitations, depending on tumor location, invasiveness of the surgery, and the amount of resected tissue; however, the movement in patients who undergo flap reconstruction is typically limited to minimize tension on the wounds and support flap survival, usually for approximately 2–3 weeks until the viability of flap is confirmed.

### Description of follow up routine

Surveillance for local recurrence include both clinical and imaging assessments (ultrasound, computed tomography [CT] and/or magnetic resonance Imaging [MRI]) every 6 months for the first 2 years, followed by annually thereafter until 10 years. For the patients with myxoid liposarcoma and low-grade fibromyxoid sarcoma, additional chest and abdominal CT are additionally taken at all visits. For other histological types, a systemic examination is performed based on the presenting symptoms. Functional imaging (e.g., positron emission tomography scan) are not routinely used in this cohort of patients.

### Variables and outcome measures

Variables included patient demographics, tumor location, diagnosis, types of surgical interventions, intraoperative details and comorbidities. In this study, upper extremity sarcomas were defined by a tumor origin between the fingers and medial border of the scapula; lower extremity sarcomas were those that originated between the toes and upper border of the iliac crest; trunk sarcomas were tumors other than upper- and lower extremity tumors [6]. Tumors arising in the retroperitoneum, chest cavity, abdominal cavity, or head–neck area, were not included.

To evaluate surgical outcomes, we examined surgical margins, duration of surgery, and length of hospital stay. Additionally, post-surgical comorbidities were investigated, with a particular focus on reoperations (return to operation room) due to wound complications (e.g., debridement of infected or necrotic tissue, operative drainage of seromas or hematomas, and wound dehiscence).

### Demographics, description of study population

Among the 132 patients included in this study, there were 60 females (45.5%) and 72 males (54.5%), with a median age of 57.1 years (range 15 to 87.5 years) at the time of diagnosis. The diagnosis was WDLS/ALT (66 patients, 50.0%), myxoid liposarcoma (31, 23.5%), leiomyosarcoma (22, 16.7%), myxofibrosarcoma (4, 3.0%), low-grade fibromyxoid sarcoma (3, 2.3%), and others. The positions of primary tumors were upper extremities in 25 patients (18.9%), lower extremities in 88 patients (66.7%) and trunk in 19 patients (14.4%). The maximum tumor dimension was under 5 cm in 67 patient (50.8%) and over 5 cm in 65 patients (49.2%). Forty patients (30.3%) underwent previous surgery, indicating tumor removal at other hospitals with inadequate margin resection or macroscopic residual tumor (Table 1). All the patients had at least 2 years of follow-up, and the median follow-up was 4.7 years (range 2 to 19 years).

### Accounting for all patients / study subjects

At our institution, pre-operative radiotherapy is administered to all patients with low-grade soft-tissue sarcomas, except for those treated by amputation, tumors of cutaneous origin (e.g., dermatofibrosarcoma protuberans), solitary fibrous tumors, or patients with metastases prior to definitive surgery. All other eligible patients during the study period were included in the analysis.

### Statistical analysis

Categorical variables were presented as frequencies and percentages and compared using the Chi-squared (χ2) test. Continuous variables were presented as medians with corresponding interquartile ranges (IQRs) or means with standard deviations. As oncologic outcomes, local recurrence-free survival, distant metastasis-free survival, and disease-specific survival were assessed. Local recurrence-free survival was defined as the period from definitive surgery to either the date of the last follow-up, or local recurrence. Distant metastasis-free survival was defined as the period from definitive surgery to either the date of the last follow-up, or distant metastasis. Disease-specific survival was defined as the period from definitive surgery to either the date of the last follow-up, or death due to disease progression. Kaplan–Meier curves were used to illustrate the probabilities of the oncologic outcomes, with univariate survival differences demonstrated using the log-rank test with hazard ratios (HRs) and 95% confidence intervals (95% CIs). Multivariable Cox regression analyses were performed using a forward stepwise selection method, including variables with *p* < 0.20 in the univariate analyses. A *p* value < 0.05 was considered statistically significant. All statistical analyses were performed using the SPSS statistical software (version 29.0; IBM, Armonk, NY, USA).

## Results

### Surgical outcomes

Among 132 patients, the number of patients who underwent plastic reconstruction was 104 (78.8%), including free flaps (68 patients, 51.5%), pedicled flaps (34 patients, 25.8%), and split-thickness skin grafts (2 patients, 1.5%; Table 2). The number of patients with a history of previous surgery was higher among those who underwent plastic reconstruction compared to those who had direct closure (38 patients, 36.5% vs. 2 patients, 7.1%; *p* = 0.003). The surgical margins were R0 in 123 (93.2%), R1 in 7 (5.3%), and R2 in 2 (1.5%). Among 104 patients who underwent plastic surgery, 100 patients (96.2%) had R0 margins and four patients (3.8%) had R1/R2 margins; among 28 patients with direct closure, 23 patients (82.1%) had R0 margins and five patients (17.9%) had R1/R2 margins (*p* = 0.009). Among the 66 patients with WDLS/ALT, the surgical margin was R0 in 59 patients (89.4%), R1 in five patients (7.6%), and R2 in two patients (3.0%).

**Table 2:** The details of definitive surgery

Character (*n* = 132)		With plastic surgery (*n* = 104)	Without plastic surgery (*n* = 28)	*p* value
Surgical margin	R0	100 (96.2%)	23 (82.1%)	0.009
	R1/2	4 (3.8%)	5 (17.9%)	
Position	Upper extremity	21 (20.2%)	4 (14.3%)	0.768
	Lower extremity	68 (65.4%)	20 (71.4%)	
	Trunk	15 (14.4%)	4 (14.3%)	
Size	Under 5 cm	52 (50%)	15 (53.6%)	0.737
	Over 5 cm	52 (50%)	13 (46.4%)	
Depth	Superficial	14 (13.5%)	3 (10.7%)	0.700
	Deep	90 (86.5%)	25 (89.3%)	
Previous surgery	No	66 (63.5%)	26 (92.9%)	0.003
	Yes	38 (36.5%)	2 (7.1%)	

The median duration of surgery was 3.9 h (IQR: 1.6–5.3) among all types of surgery; the median durations of surgery for plastic reconstruction and direct closure were 4.2 (IQR: 1.7–5.9) and 2.2 (IQR: 1.3–4.9) hours, respectively. The median duration of hospitalization was 9 (IQR: 7–16) days among all types of surgery; the median durations of hospitalization among patients who underwent plastic reconstruction and direct closure were 10 (IQR: 7–16) and 8 (IQR: 5–10) days, respectively.

### Oncologic outcomes

**Fig. 1:**
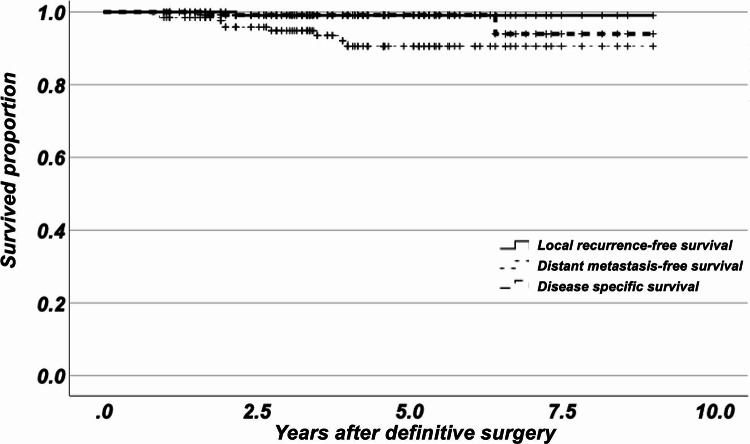
Kaplan–Meier curves for oncologic outcomes. The straight line indicates local recurrence-free survival, the dotted line indicates distant metastasis-free survival, and the broken line indicates disease-specific survival

The Kaplan–Meier curves revealed a 5-year local recurrence-free survival of 99.1% (95% CI: 97.3–100); there was one instance of local recurrence after R2 resection with plastic reconstruction of WDLS/ALT on the chest wall. The 5-year distant metastasis-free survival was 90.6% (95% CI: 84.5–96.7). We identified nine metastases during follow-up: six patients with myxoid liposarcoma; one patient with WDLS/ALT with biopsy-proven dedifferentiated liposarcoma at the metastatic sites without local recurrence; one patient with leiomyosarcoma; and one patient with malignant peripheral nerve sheath tumor. The 5-year distant metastasis-free survival of the patients with WDLS/ALT was 98.2% (95% CI: 94.9–100) and those with myxoid liposarcoma was 74.6% (95% CI: 56.4–92.8). The univariate analysis for distant metastasis-free survival revealed that myxoid liposarcoma was a negative prognostic factor (HR: 6.38, 95% CI: 1.58–25.49; *p* = 0.009), while WDLS/ALT was a positive prognostic factor (HR: 0.12, 95% CI: 0.02–0.99; *p* = 0.049; Table 3). The 5-year disease-specific survival was 99.2% (95% CI: 97.6–100%; Fig. 1).

**Table 3:** The univariate analysis for distant metastasis

Character (*n* = 132)	Hazard ratio	95% CI	*p* value
Gender (Female vs Male)	0.33	0.07–1.59	0.165
Histology			
Myxoid liposarcoma versus Others	6.38	1.59–25.49	0.009
WDLS/ALT versus Others	0.12	0.02–0.99	0.049
Position (Lower extremity vs Others)	0.95	0.24–3.84	0.940
Size (Over 5 cm vs Under 5 cm)	0.85	0.23–3.15	0.803
Depth (Superficial vs Deep)	0.45	0.14–1.40	0.166
Previous surgery (Yes vs No)	1.89	0.51–7.03	0.345
Surgical margin (Wide vs Others)	0.64	0.08–5.10	0.671
Plastic surgery (Yes vs No)	2.48	0.31–19.89	0.392

### Complications

Among 132 patients, reoperations due to wound complications occurred in 28 (21.2%) patients; 6 patients (21.4%) out of 28 patients with direct closure and 22 patients (21.2%) out of 104 patients with plastic reconstruction (*p* = 0.705). The reasons for reoperation in patients with direct closure were seroma/hematoma in one patient (3.6%), wound dehiscence in two patients (7.1%), and infection in three patients (10.7%). In contrast, among the patients with plastic reconstruction, reoperations were required due to seroma/hematoma in six patients (5.8%) (five patients with free flap, one patient with skin grafts), flap necrosis in seven patients (6.7%) (two patients with pedicled flap, five patients with free flap), infection in eight patients (7.7%) (four patients with pedicled flap, four patients with free flap), and wound dehiscence in one patient (1.0%) (with free flap). The percentage of reoperation for other various factors is shown in Table 4.

**Table 4:** The factors associated with reoperation after definitive surgery

Character		Number (*n* = 132)	Reoperation (*n* = 28)	Percentage	*p* value
Reconstruction	Direct closure	28	6	21.4%	0.734
	Pedicled flap	34	6	17.6%	
	Free flap	68	15	22.1%	
	Skin graft	2	1	50.0%	
Position	Upper extremity	25	4	16.0%	0.436
	Lower extremity	88	18	20.5%	
	Trunk	19	6	31.6%	
Size	Under 5 cm	67	13	19.4%	0.606
	Over 5 cm	65	15	23.1%	
Depth	Superficial	17	5	29.4%	0.376
	Deep	115	23	20.0%	

### Histological necrosis

**Fig. 2:**
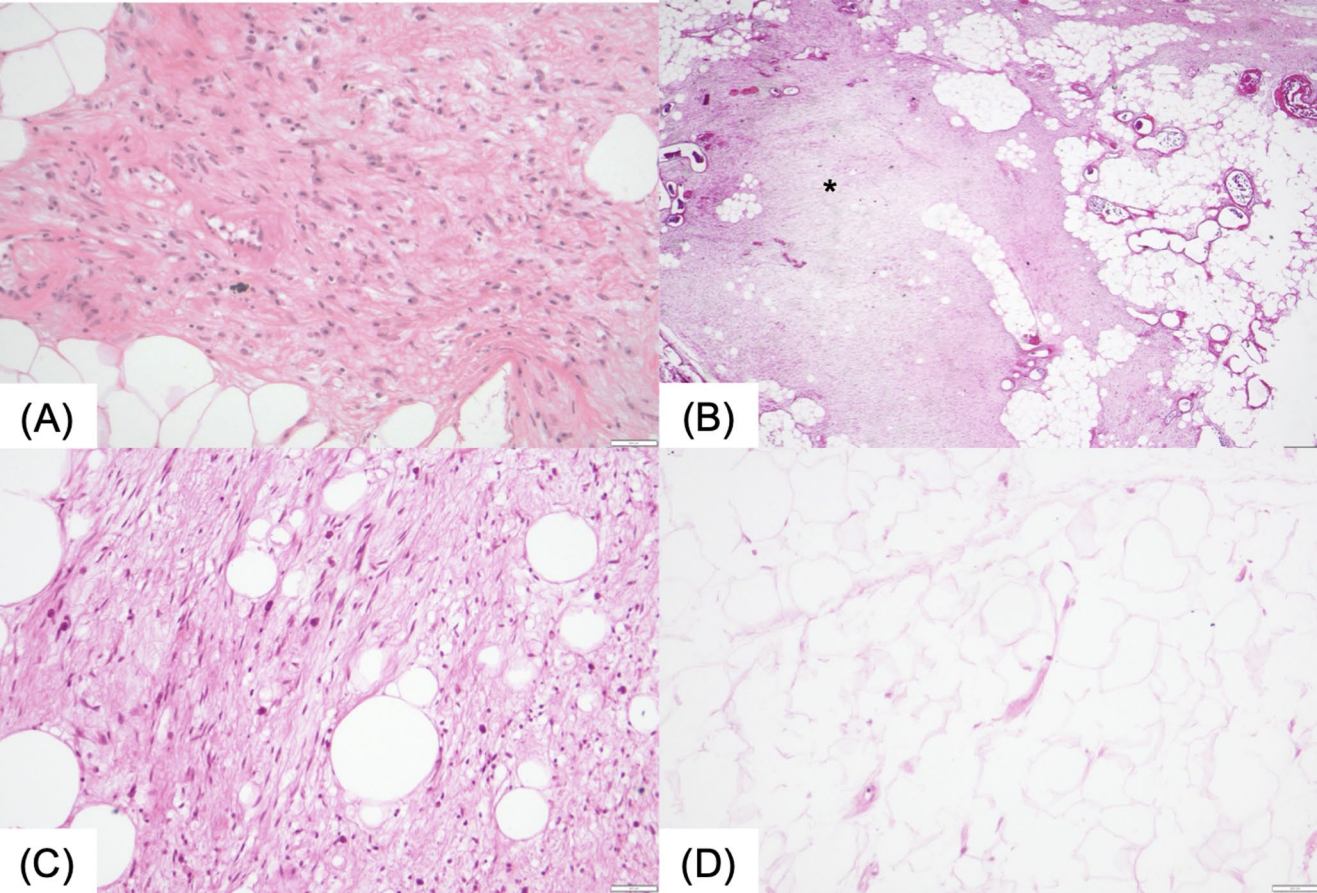
A representative patient with atypical lipomatous tumor showing a good pathological response. The patient (80-year-old female) was diagnosed with atypical lipomatous tumor in the left thigh via biopsy (**A**, Haematoxylin and eosin staining, 200× magnification). After pre-operative radiotherapy, the tumor showed an extensively necrotic area (*); approximately 90% of the tumor was considered to be necrotic (**B**, Haematoxylin and eosin staining, 100× magnification). Several septa contain spindle cells with atypical enlarged hyperchromatic nuclei and some small clusters of atypical adipocytes (**C**, Haematoxylin and eosin staining, 200× magnification). The fatty tissue exhibits a fatty structure without the presence of adipocytes (**D**, Haematoxylin and eosin staining, 200× magnification)

**Fig. 3:**
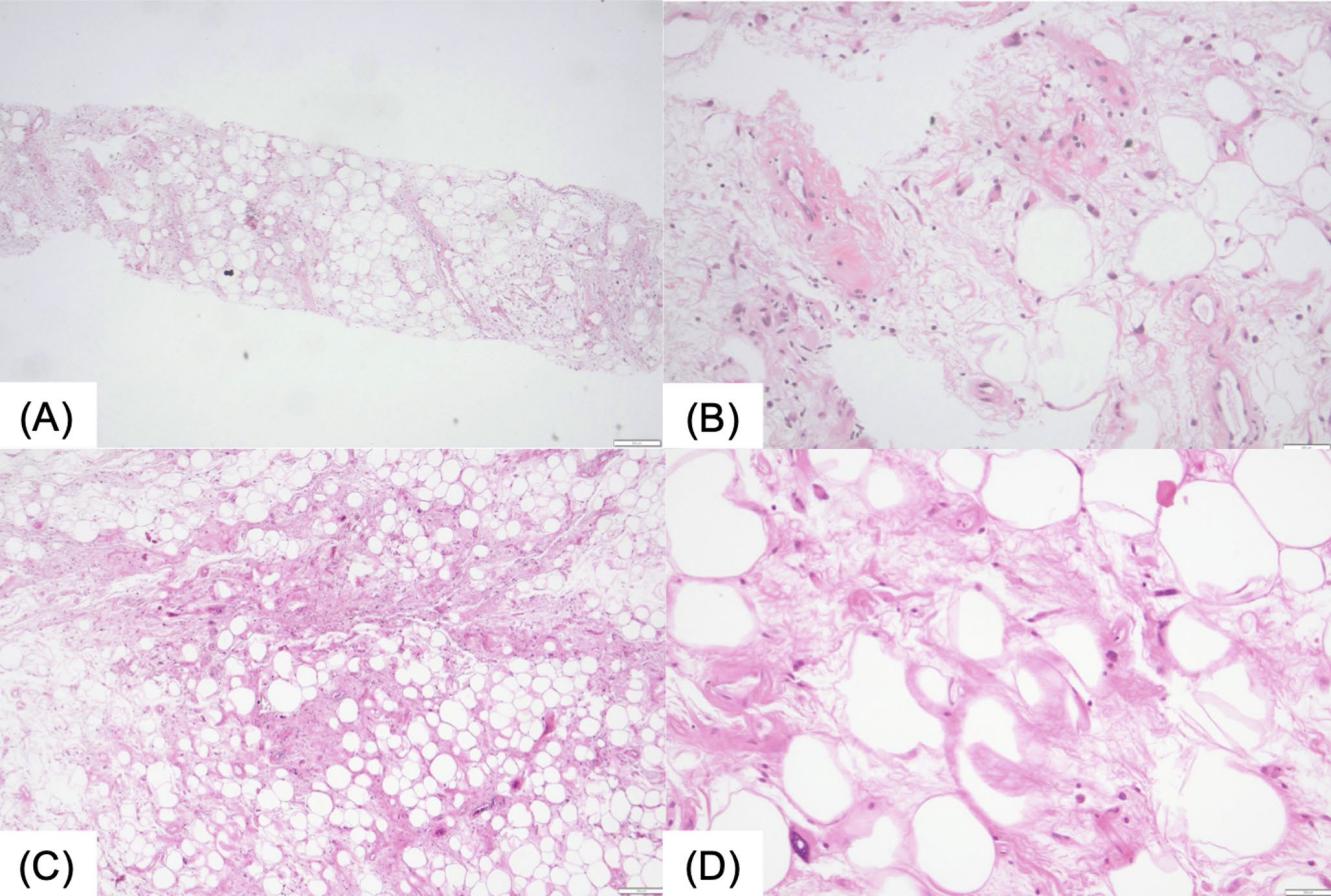
A representative patient with atypical lipomatous tumor showing a poor pathological response. The biopsy specimen (61-year-old male, right thigh) showed an atypical structure of fatty tissue with the presence of atypical stromal cells and fibrous septa (**A**, Haematoxylin and eosin staining, 50× magnification; **B**, Haematoxylin and eosin staining, 200× magnification). After pre-operative radiotherapy, the atypical structure of the fatty tissue with the presence of atypical stromal cells remained, with an almost 10% necrotic area (**A**, Haematoxylin and eosin staining, 100× magnification; **B**, Haematoxylin and eosin staining, 200× magnification)

The histological necrosis rates of the resected specimens were available in 58 patients. The median necrosis rates were 60% (IQR: 25–90%) among all patients, 35% (IQR: 10–63%) among the 30 patients with WDLS/ALT, and 95% (IQR: 75–100%) among the 21 patients with myxoid liposarcoma (Figs. 2 and 3).

## Discussion

Soft-tissue sarcoma can be locally infiltrative, with the possibility of microscopic residue around the surgical bed [14]. Surgery without any pre-operative therapies might be reasonably considered only for the patients whose margin status is expected to be unequivocally negative and enough wide to encompass the reactive zones around the tumor boundary [15]. Before the adoption of pre-operative therapy, patients with major neurovascular or multicompartmental invasion were primarily considered for amputation to ensure adequate surgical margins. However, now pre-operative radiotherapy is recommended as a standard choice for stage II or higher soft-tissue sarcomas in such conditions [15].

Despite the lack of evidence of pre-operative radiotherapy for the low-grade soft-tissue sarcomas, the efficacy of post-operative radiotherapy in patients who underwent limb-sparing surgery has been proven through a randomized prospective trial with 141 patients [16]. Among them, subgroup analysis with 50 patients with low-grade soft-tissue sarcoma was performed, and the results indicated a significant improvement in local recurrence-free survival among the patients with post-operative radiotherapy (*p* = 0.016) [16]. A meta-analysis, including 408 patients with low-grade soft-tissue sarcoma, showed a favoring trend among the patients who received pre-operative radiotherapy than patients with no radiotherapy in local recurrence (odds ratio = 0.38, *p* = 0.11) [17]. These results suggest that some patients with low-grade soft-tissue sarcoma may benefit from pre-operative radiotherapy.

The primary limitation of this study is the presence of confounding factors, which may have affected the outcomes. Since pre-operative radiotherapy was combined with wide excision and flap reconstruction, supported by plastic surgeons, it may be difficult to attribute our results solely to the benefit of pre-operative radiotherapy. Secondly, due to the possible underestimation of tumor grade in the biopsy specimens, the diagnosis of low-grade soft-tissue sarcoma may be dubious. Thirdly, the follow-up period may have affected the results of our study. Although most local recurrences among patients with low-grade soft-tissue sarcoma occurred within 5 years [18], the possibility of late-onset local recurrence cannot be ruled out. However, as the exclusive sarcoma treatment center in the area, our hospital is routinely notified of any oncologic event –including local recurrence or distant metastasis– by primary medical services. The circumstance likely helps to minimize detection bias for local recurrence.

We analyzed the outcomes of 132 patients with low-grade soft-tissue sarcoma treated with pre-operative radiotherapy. Adequate margin status (R0) was achieved in 123 patients (93.2%), and the local recurrence-free survival was 99.1%. Generally, marginal excision is associated with the risk of leaving tumor cells in the surgical bed, which may predispose the patient to local recurrence [19]; however, planned marginal excision may be indicated in selected patients with the aim of preserving functionality [19]. The decision should therefore be based on the diagnosis, with careful consideration of the balance between morbidity and mortality associated with recurrence—especially for tumors with a low risk of local recurrence [19].

Regarding WDLS/ALT, a representative tumor of low-grade soft-tissue sarcomas, marginal resection is widely indicated in many institutions [20]. This is because, when local recurrence does occur, it is often treatable via repeat resection with acceptable morbidity and functional outcomes [21]. A retrospective study at the Instituto Ortopedico Rizzoli involving 43 patients with WDLS/ALT treated by marginal resection reported six patients with local recurrence (13.9%, mean latency from prior surgery: 48 [33–96] months). Of these, five patients were treated via re-excision with marginal margins, with no evidence of further local re-recurrence, while one patient experienced multiple local recurrences culminating in dedifferentiation [22]. A systematic review of 18 studies involving 793 patients with WDLS/ALT reported that the local recurrence rate was 11.9% (69/580 patients) among the 580 patients who underwent marginal excision, and 3.3% (7/213 patients) in patients who underwent wide resection [19]. In this study, we experienced one local recurrence after R2 resection among 66 patients with WDLS/ALT; the local recurrence rate was 0% in patients with both R0 and R1 margins. Although direct comparisons to previous studies [19, 22] are difficult, a potential positive impact of pre-operative radiotherapy on local control may be expected for the reduction of local failure. Whether this improvement in local control is of clinical value remains undetermined, given the increased risk of peri-operative complications, as well as the cost and complexity of plastic reconstruction.

In this study, the 5-year distant metastasis-free survival was 90.6% (95%CI, 84.5–96.7) and the 5-year disease specific survival was 99.2% (95%CI, 97.6–100%). A study analyzed patterns of sarcoma-specific death in low-grade soft-tissue sarcomas, classifying causes of death as died from locally recurrent disease or died from distant disease [23]. Among 2041 patients aged ≥ 16 with low-grade soft-tissue sarcoma at all sites, 181 (9%) died from disease: among them, 105 patients (58%)  died from locally recurrent disease, 59 [32%] died from distant disease, and others [23]. Died from locally recurrent disease occurred predominantly in the retroperitoneum (80%) and rarely in the extremity (3%), whereas died from distant disease occurred across various locations (extremity [47%], trunk [18%]) [23]. Dedifferentiation of WDLS/ALT, which is associated with potential for distant metastasis, has been reported at 1%–4% [24, 25]. In this study, we experienced one patient (1.5%) with biopsy-proven dedifferentiation at metastatic site out of 66 patients with WDLS/ALT. It is difficult to conclude from this study whether improved local control with preoperative radiotherapy reduces the incidence of dedifferentiation and improves the oncologic outcomes.

Pre-operative radiotherapy could increase the risks of both acute post-operative wound complications (e.g., skin necrosis, oedema) and late toxicities (e.g., fibrosis, pathologic fractures, or joint contractures) [26]. A randomized study performed by O’Sullivan et al. [4] revealed a higher rate of wound complications in patients who underwent pre-operative radiotherapy (31/88 patients [35%] vs 16/94 patients [17%], pre-operative vs. post-operative, respectively; *p* = 0.01). Likewise, a meta-analysis demonstrated similar tendencies in wound complications, seen more frequently in pre-operative than post-operative radiotherapy (OR: 2.92; *p* < 0.0001) [17]. To mitigate wound complications, plastic reconstruction for wound closure is employed to lessen the tension of the wound and fill the dead space [6, 8]. In a retrospective series of 82 patients, the complications of plastic reconstruction were compared with those of direct closure for irradiated wounds; it was revealed that direct closure–used in earlier period in the series–had a higher rate of wound complications (51% vs 19%) and reoperations (35% vs 10%) compared to plastic reconstruction [27]. However, no randomized trials have specifically evaluated the plastic reconstructions for wound closure. In this study, there was no difference in the rate of reoperation between those who underwent plastic reconstruction and direct closure due to wound complications (24.0% vs 20.6%, *p* = 0.705). This is because we selected direct closure for wounds unlikely to be under excessive tension, while wounds with a high risk of failure were treated with plastic reconstruction, which can mask the potential benefits of plastic reconstruction.

A retrospective study with patients of 50 patients with soft-tissue sarcomas revealed that preoperative radiotherapy (50 Gy) induced 50% histological necrosis in all grades of soft-tissue sarcoma and 67.5% histological necrosis in low-grade soft-tissue sarcoma [28]. In this study, we observed the good histological response to pre-operative radiotherapy, especially in the patients with myxoid liposarcoma. Myxoid liposarcoma is considered to be a radiosensitive tumor, and it is hypothesized that radiotherapy predominantly damages the capillary rich vascular network within the tumor [29]. In a single-center series of 329 patients with localized myxoid/round cell or pleomorphic liposarcoma treated with surgery over 25 years, local recurrence rates were 25% [30]. In the multivariable analysis, additional radiotherapy was independent prognostic factors for local recurrence-free survival (HR = 0.39, 95% CI 0.23–0.65, *p* = 0003) [30]. While a standard radiation dose of approximately 50 Gy is usually given for soft-tissue sarcoma [31], a single-arm phase II study (Dose Reduction in Myxoid Liposarcoma, DOREMI study) assessed the radiological effects of 36 Gy of preoperative radiotherapy in patients with primary non-metastatic myxoid liposarcoma [32]. This study showed 100% local control and a median change of the tumor volume was − 60% [32] and histological analysis revealed that the percentage of viable tumors in resected specimen was 7.5% (IQR 5–15%) [29]. Moreover, the radiotherapy to myxoid liposarcoma might induce differentiation via maturation of adipocytes, originally described after treatment with trabectedin [33, 34]. A phase II study combining pre-operative trabectedin and radiotherapy (45 Gy) reported that five out of 39 patients (13%) achieving a complete histological response, and 20 out of 39 patients (51%) having over 90% histological necrosis [35]. Since our results also demonstrated a good histological response, it suggested the pre-operative radiotherapy should be considered for myxoid liposarcoma even with lower-grade patients.

In conclusion, pre-operative radiotherapy for low‑grade soft‑tissue sarcoma was associated with favorable local‑control outcomes. However, because of the risk of peri-operative complications, pre-operative radiotherapy should be reserved for patients most likely to benefit and used selectively administered for tumors at high risk of positive or marginal margins (e.g., those adjacent to major nerves, vessels or enveloping critical structures) or for highly radiosensitive subtypes. Small, superficially located tumors are generally exempt. The efficacy and invasiveness of this treatment should be carefully balanced based on the patients’ individual backgrounds and the consensus of multidisciplinary team.

## Data Availability

Data will be made available on reasonable request.
